# No evidence that ‘fast-mapping’ benefits novel learning in healthy Older adults

**DOI:** 10.1016/j.neuropsychologia.2014.05.011

**Published:** 2014-07

**Authors:** Andrea Greve, Elisa Cooper, Richard N. Henson

**Affiliations:** MRC Cognition & Brain Sciences Unit, 15 Chaucer Road, Cambridge CB2 7EF, England, United Kingdom

**Keywords:** Fast mapping, Episodic encoding, Aging, Hippocampus, MRI

## Abstract

Much evidence suggests that the Hippocampus is necessary for learning novel associations. Contrary to this, Sharon, Moscovitch, and Gilboa (2011) reported four amnesic patients with Hippocampal damage who maintained the capacity to learn novel object-name associations when trained with a ‘fast-mapping’ (FM) technique. This technique therefore potentially offers an alternative route for learning novel information in populations experiencing memory problems. We examined this potential in healthy ageing, by comparing 24 Older and 24 Young participants who completed a FM procedure very similar to Sharon et al. (2011). As expected, the Older group showed worse memory than the Young group under standard explicit encoding (EE) instructions. However, the Older group continued to show worse performance under the FM procedure, with no evidence that FM alleviated their memory deficit. Indeed, performance was worse for the FM than EE condition in both groups. Structural MRI scans confirmed reduced Hippocampal grey-matter volume in the Older group, which correlated with memory performance across both groups and both EE/FM conditions. We conclude FM does not help memory problems that occur with normal ageing, and discuss theoretical implications for memory theories.

## Highlights

• ‘Fast mapping’ does not benefit novel learning in healthy Older adults.
• Older and Younger healthy adults show worse memory under ‘Fast Mapping’.
• Older and Younger healthy adults show better memory for Episodic Encoding.
• Older adults show memory decline linked to Hippocampal grey-matter volume loss.
• Fast mapping and episodic encoding are correlated with Hippocampal grey-matter.

## 1 Introduction

Lesions to the medial temporal lobe (MTL), particularly those that include the Hippocampus, are known to produce amnesia; particularly deficits in episodic memory (Scoville & Milner, 1957). It has been suggested that Hippocampal lesions specifically affect the ability to rapidly encode new associations between two items, such as the name of a novel object; a key feature of declarative, relational and recollective memory theories (Rempel-Clower, Zola, Squire, & Amaral, 1996; Spiers, Maguire, & Burgess, 2001; Zola-Morgan, Squire, & Amaral, 1986). Nonetheless, some amnesic patients show evidence of learning new associations when this information is linked to information established prior to the onset of amnesia (O’Kane, Kensinger, & Corkin, 2004; Skotko, Kensinger, Locascio, Einstein, & Rubin, 2004), albeit at a slower rate than controls (Bayley & Squire, 2005). Moreover, evidence from individuals with Hippocampal damage at birth (developmental amnesia) suggests that they can learn new associations, at least to the extent that they acquire relatively normal levels of semantic knowledge despite their impaired episodic memory (Martins, Guillery-Girard, Jambaqué, Dulac, & Eustache, 2006; Vargha-Khadem, Gadian, & Mishkin, 2001). This intact associative learning in developmental amnesia is consistent with claims that the brain has two, complementary learning systems, with rapid learning occurring in the Hippocampus (as necessary for episodic memory) and slower learning occurring in other cortical regions (to enable semantic memory). Some computational models justify the slower cortical learning in terms of minimising interference between competing associations, as they become integrated into semantic memory (McClelland, McNaughton, & O’Reilly, 1995; Norman & O’Reilly, 2003).

Evidence from healthy young children in the developmental literature suggests that new associations can be learned very quickly. Surprisingly, these associations seem to be incorporated directly into their developing semantic memory, without needing a period of time for slow cortical learning. For example, it has been claimed that children as young as 18 months can rapidly associate a novel word with a novel object, and then continue to demonstrate semantic knowledge and comprehension of that word in future behaviour; a phenomenon called ‘fast-mapping’ (Carey & Bartlett, 1978). Such fast-mapping may account for the massive increase in vocabulary during the first few years of life (Bion, Borovsky, & Fernald, 2013). It is not clear whether this rapid learning is specific to the developing brain, or reflects instead computational factors such as reduced interference from existing associations. Nonetheless, the finding that individuals with developmental amnesia following Hippocampal damage appear to have normal vocabulary acquisition raises the possibility of a rapid cortical learning mechanism that does not depend on the Hippocampus.

As mentioned previously, one possibility is that cortical learning can be rapid when new information is presented in conjunction with familiar information, and this provides a schema for assimilating the new information (Tse, Langston, Kakeyama, Bethus, & Spooner, 2007; van Kesteren, Ruiter, Fernández, & Henson, 2012). This is consistent with demonstrations that fast-mapping in children is more likely when the novel word and novel object are present together with other familiar objects (Halberda, 2006; Kucker & Samuelson, 2012). The familiar object may activate a schema, which in turn helps discriminate or individuate the novel item, facilitating its integration into semantic memory. If so, then FM may not be unique to children, and might occur in adults under the appropriate conditions.

**Fig. 1:**
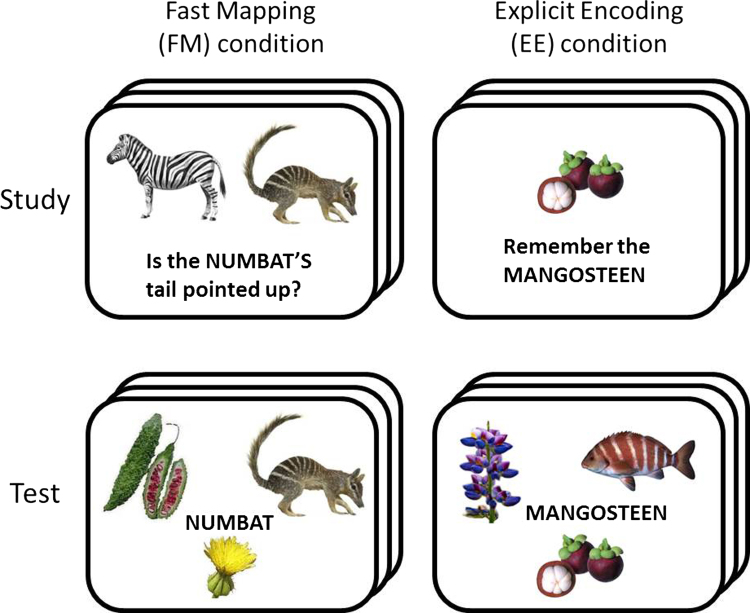
Example trials for the Study and Test phases of the fast-mapping (FM) and explicit encoding (EE) conditions.

This was part of the rationale for a recent study by Sharon, Moscovitch, and Gilboa (2011), which investigated FM in four adults with acquired amnesia following damage to the MTL that included the Hippocampus in every case. Amazingly, despite their amnesia on typical episodic memory tests, performance on a paired associate learning task was restored to the level of matched controls when a fast-mapping procedure was used. More specifically, Sharon et al. (2011) tested participant׳s ability to learn the association between a novel word and a novel picture of an object (e.g., an animal or fruit). They compared two conditions: a standard intentional learning condition they called explicit encoding (EE) and an incidental learning condition they called the fast-mapping (FM) condition. In each trial of the study phase of the EE condition, participants were presented with one word and one object, and told to remember the name of the object. In each study trial of the FM condition, on the other hand, participants were presented with two objects – one novel and one familiar – and answered a yes/no question that employed a novel word to refer to the novel object (such that participants had to infer that the word was the name of the novel object; see Fig. 1). Thus the FM condition differed from the EE condition in terms of (i) involving incidental rather than intentional encoding, (ii) presenting a concurrent familiar object, and (iii) requiring a response based on the disjunctive inference needed to infer the name of the novel object. Following each study phase, identical test phases were completed, where participants were shown a single word together with three objects, and asked to select the object that was paired with the word in the study phase (i.e., 3-alternative forced choice, 3AFC). Memory was tested at two delays: after 10 min and after 1 week. Regardless of delay, the 3AFC performance showed a striking interaction between FM vs. EE and patient vs. control group, such that controls performed better on the EE than FM condition, and patients performed better on the FM than EE condition (see Smith et al., 2014, and Section 4). Most importantly, the patients were no longer “amnesic” in the FM condition, i.e., performed at a similar level to controls. Furthermore, two additional patients who had damage that included the Anterior Temporal Lobe (ATL) did not show the same improvement with FM as did the other patients, implicating the ATL in this form of rapid but non-Hippocampal associative learning. These findings clearly offer much hope for the rehabilitation of people with memory problems, in that the fast-mapping procedure may help individuals with significant Hippocampal atrophy to acquire new information.

Given that the Hippocampus has also been shown to decrease in volume during normal, healthy ageing (Du, Schuff, Chao, Kornak, & Jagust, 2006; Jernigan, Archibald, Fennema-Notestine, Gamst, & Stout, 2001; Raz, Rodrigue, Head, Kennedy, & Acker, 2004; Schuff et al., 1999), and that older people generally perform worse on tests of associative memory than do younger people (Naveh-Benjamin, Guez, Kilb, & Reedy, 2004; Naveh-Benjamin, Hussain, Guez, & Bar-On, 2003), we wondered whether FM could also be used to support memory in older individuals. Previous FM studies have almost exclusively focused on young children, with only a few studies investigating university students (Halberda, 2006, Markson & Bloom, 1997). While Sharon et al. (2011) were the first to extend FM studies into a middle-aged population, the present study is the first to investigate FM learning in Older adults, and directly compare results with Young adults. More specifically, we replicated Sharon et al.׳s design (bar a few procedural changes, considered later) on a group of 24 older people with average age of 66, and a group of 24 younger participants with average age of 27. The same participants also underwent a structural MRI scan so that we could estimate the volume of their Hippocampi and ATL, and relate these volumes to their behavioural performance on the FM and EE tasks. The primary aim of our study was to see whether FM alleviated the relative memory deficit for Older vs. Young groups, i.e., to test for an interaction between FM and EE conditions and age group, while a secondary aim was to see whether FM and EE had differential dependencies on Hippocampal and ATL volumes.

## 2 Methods

### 2.1 Participants

24 Young (aged 18–42, mean of 27; 16 females) and 24 older (aged 55–79, mean of 66; 14 females) individuals were recruited from the volunteer panel of the MRC Cognition and Brain Science Unit, allowing full-counterbalancing of stimuli within each group. All participants reported normal or corrected-to-normal visual acuity, provided informed consent and received monetary compensation for participation, as approved by a local ethics committee (Cambridge Psychological Research Ethics Committee reference 2005.08).

### 2.2 Stimuli

Stimuli consisted of 2 sets of coloured pictures of 24 unfamiliar and 24 familiar animals, fruits, vegetables and flowers, approximately matched for perceptual qualities by Sharon et al. (2011). Note that the unfamiliar objects and their names were in fact real, but just extremely unlikely to have been encountered before by the participants (owing to their rarity or absence in the participants׳ culture). Nonetheless, because the population tested here originated from a different cultural background than those tested by Sharon et al. (2011), who were from Israel, we conducted a pilot study to prune the stimuli to conform to the cultural knowledge of our British participants (see Appendix A for a detailed list of changes). Assignment of stimulus sets to the EE and FM conditions was counterbalanced across participants.

### 2.3 Procedure

**Fig. 2:**
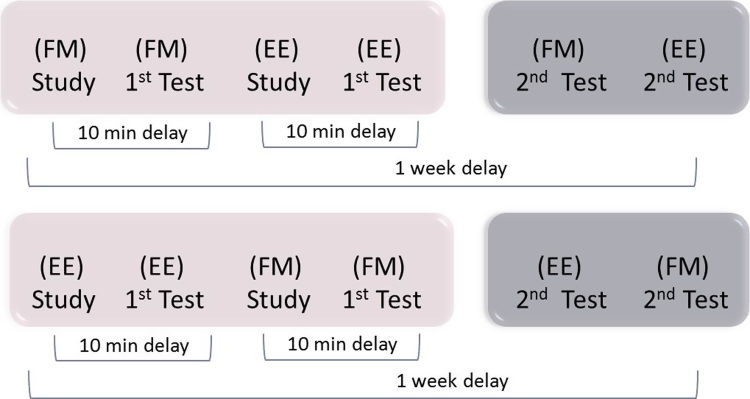
Experimental design showing the order of Study and Test phases, and the two possible task orders for fast-mapping (FM) and explicit encoding (EE) conditions.

Fig. 2 illustrates the experimental design. Participants were tested during 2 visits, one week apart. In the first visit, they performed the study and immediate (10 min) test phases of the FM followed by EE conditions; during the second visit, they performed the delayed (1 week) test phase. For the Older group, the order of EE and FM tasks was counterbalanced (with 12 participants doing each order, i.e., FM, then EE, and EE followed by FM). The Young group on the other hand performed the tasks in the same order as Sharon et al. (2011), i.e., FM tasks before EM tasks. This issue of task order is addressed in Section 4.

### 2.4 Study phase

The study phase for each condition, i.e., FM and EE, started with a practice phase that familiarised participants with the study procedure for that condition, using items unique to the practice. In the fast-mapping (FM) condition, an unfamiliar and familiar item were presented on a computer screen (see Fig. 1), both with an equal likelihood of left/right positioning across trials. Participants were told press one of two keys in order to respond yes/no to a question regarding a perceptual property of the unfamiliar item (e.g., “Is the Numbat׳s tail pointing up?”). The question was both printed on the screen and presented aurally via headphones. As the FM condition is an incidental learning task, participants were informed that the task was one concerning object perception, and were not informed of the interest in ‘learning’ or the item memory test that was to follow. In the explicit encoding (EE) condition, a single unfamiliar object was presented in the centre of the screen, and participants received visual and auditory instruction to remember the name of the object (e.g., “Remember the Numbat”).

Both FM and EE study trials began with a fixation cross presented for 500 ms, which was followed by the simultaneous visual and aural presentation of the relevant instruction for 3000 ms, followed by the visual object(s) for 3500 ms. Each object-name pair was presented a second time at random intervals (i.e., two study presentations). For the FM condition, the target object switched sides between its initial and repeated presentation, and was presented in conjunction with a different question that still used the object׳s name.

In the FM condition, participants indicated their ‘yes’/‘no’ decision to the instruction by pressing one of two keys on a keyboard with the index finger of their left hand and index finger of their right hand. Half of the answers were designed to require a ‘yes’ and the other half ‘no’ response. The mapping of yes/no to keys was counterbalanced across participants. Participants were instructed to prioritise accuracy over speed, but nonetheless had to provide a response within the 3500 ms time window. If a response was not given within time limit, then the programme prompted the participant for a response. No response was required in the EE condition.

### 2.5 Test phase

Each study phase was followed by a 10 min retention interval, during which participants performed a distractor task to prevent rehearsal. The distractor task was one of the four quarters of the Cattell Culture Fair Scale 2 intelligence test (Cattell, Krug, & Barton, 1973), similar to Raven׳s matrices – such that we could also estimate each participant׳s general intelligence ‘g’ by summing their Cattell test scores across the 4 intervals.

The test phase resumed with 24 trials that assessed memory for all 24 studied objects using a three-alternative forced choice (3AFC). FM and EE learning were tested with identical procedures: a previously-presented word was shown in the middle of the screen, surrounded by three alternative objects (Fig. 1). Participants were instructed to select the object that was previously paired with the word by pressing one of three keys on the keyboard using their right hand.

Test trials started with a fixation cross for 500 ms, followed by the display of a word and three choice objects, which remained on the screen until participants pressed a key. Note that each object was shown in three separate test trials: once as a target and twice as a foil, with the item appearing once in each of the 3 locations. Location of the target was balanced so that targets were equally likely to occur in any of the three locations. Considering the increased number of test trials, we presented proportions of lure trials closely matched to Sharon et al. (2011): 3 (=6%) trials with same lure, 23 (=48%) with one lure being the same category and 22 (=46%) trials with both lures of a different category.

A second testing session was conducted following a delay of one week. Test procedure and items were identical to the first test session (i.e., memory for the same object-name associations was tested). After the final 3AFC test was completed, objects were presented once more one-at-a-time, and participants rated on a 3-point scale how familiar they were with the item before the experiment. Objects for which subjects reported pre-experimental knowledge were excluded from further analysis for that individual.

### 2.6 MRI image acquisition and processing

T1-weighted structural images with 1×1×1 mm voxel size were acquired for each participant using Magnetisation Prepared Rapid Gradient Echo (MPRAGE) on a 3T TIM Trio system (Siemens, Erlangen, Germany). These images were processed in FreeSurfer (FS) (Version FreeSurfer-Linux-centos6_×86_64-stable-pub-v5.3.0, http://surfer.nmr.mgh.harvard.edu/). The automated procedure for volumetric measures of subcortical regions have been previously described by Fischl et al. (2002). This procedure segments and labels unique structures based on probabilistic information were estimated from a manually labelled training set. In brief, FreeSurfer uses a Bayesian segmentation procedure that takes into account the prior probability of a given tissue class occurring in a specific atlas location, the likelihood of the image given the tissue class and the probability of the local spatial configuration of labels given the tissue class. Total intracranial volumes (TIV) were calculated to ensure that regional brain volume analyses are not biased by potential changes in TIV.

Given our predictions in Section 1, grey-matter volume (GMV) was estimated for two *a priori* regions of interest (ROIs): sum of left and right Hippocampi, and sum of left and right Temporal Pole, or what we call here (ATL) to follow Sharon et al. (2011).

## 3 Results

We start by characterising differences between our two age groups, particularly to confirm Hippocampal atrophy in our Older relative to Young group. We then report group differences in behavioural scores in the FM and EE conditions, before regressing individual behavioural scores against general intelligence and grey-matter volume in our ROIs.

### 3.1 Brain differences

Table 1 shows MRI volume estimates for the two bilateral ROIs (Hippocampus and ATL), as well as the total intracranial volume (TIV). As expected, the Older group showed significant reductions in Hippocampal volume (*T*=3.92, *p*<.001), even when co-varying out differences in TIV (*T*=3.02, *p*=.002). No differences in ATL ROI reached significance (*T*=1.21, *p*=.23), even when co-varying out differences in TIV (*T*=1.42, *p*=.16).

### 3.2 IQ differences

Results of the Cattell IQ test showed a significantly higher estimate of general intelligence in the Young than Older group (Table 1), *T*(46)=5.65, *p*<.001, consistent with many other studies of ageing (Deary, 2013, Dickinson & Hiscock, 2010, Horn & Cattell, 1966). We address the possible influence of general intelligence in the multiple regressions performed later.

### 3.3 EE and FM performance

Only the FM condition provided behavioural data during Study, in relation to yes/no question posed about the two objects (Fig. 1). Performance was on average 94.5% accurate in the Young group and 92.4% in the old group (ranging from 78 to 100%), demonstrating good task comprehension in both groups. More importantly, there was no reliable evidence that the older participants were less able to perform this task than their younger counterparts [*T*(46)=1.59, *p*>.05, after arcsin transform to reduce skewness].

**Fig. 3:**
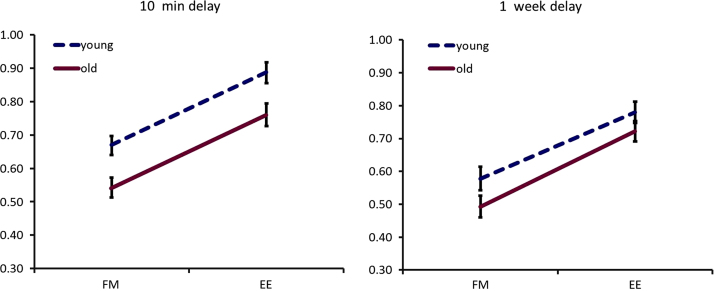
Mean 3AFC performance of Young (blue dashed line) and Older (red solid line) groups following FM and EE encoding conditions, assessed by 3AFC following a 10 min study-test delay (left panel) or a one week study-test delay (right panel). (For interpretation of the references to colour in this figure legend, the reader is referred to the web version of this article.)

Mean 3AFC accuracy during the critical test phase is shown for each age group, condition and delay in Fig. 3. A three-way analysis of variance (ANOVA) revealed three significant main effects. The main effect of Age [*F*(1,46)=8.46, *p*<.01] reflected worse memory for Older than Young groups, as expected; the main effect of Delay [*F*(1,46)=25.2, *p*<.001] reflected worse memory after 1 week than 10 min, as expected; most importantly, the main effect of Condition [*F*(1,46)=112, *p*<.001] reflected worse memory in the FM than EE condition. The two-way interaction between Age and Delay approached significance [*F*(1,46)=3.78, *p*=.06], but more importantly, none of the remaining interactions, which involved the Condition factor, approached significance [*Fs*(1,46)<1]. In other words, there was no evidence that the difference between EE and FM performance differed according to Age (or Delay).

Given that Delay did not interact with Condition, and its lack of present theoretical interest (Sharon et al. did not find any effects of retention interval either), we averaged across both Delays in all subsequent analyses. To confirm the advantage of EE over FM in both age groups, we performed follow-up, two-tailed *T*-tests, which were significant for both Young [*T*(23)=6.60, *p*<.001] and Older [*T*(23)=8.68, *p*<.001] groups. Most importantly, unlike the lack of significant difference between patients and controls in the FM condition of Sharon et al. (2011), the present Young group performed significantly better than the Older group in the FM condition [*T*(46)=2.75, *p*<.01], as well as in the EE condition [*T*(46)=2.26, *p*<.05].^1^

### 3.4 Task order

The influence of task order was tested in the Older group in which task order was counterbalanced (see Fig. 2). An ANOVA showed no significant main effect of this Order factor, nor evidence that it interacted with the Delay or Task factors (all *Fs*<1). We also restricted the full analysis to those participants who performed the FM task first, i.e., half of the Older group and the whole of the Young group, to match the Sharon et al. study. In this case, the main effects of Group, Lag and Task remained significant, *F*(1,34)>5.40, *p*<.05, but there was still no sign of any interactions involving the Task factor, all *Fs*<1, as with the main analyses above.^2^

### 3.5 Bayesian statistics

In contrast to the interaction between groups (patients and controls) and conditions (FM and EE) reported by Sharon et al. (2011), our analysis showed no evidence of differential effects of FM and EE encoding for Older relative to young participants. As with any form of classical null-hypothesis testing however, absence of evidence is not evidence of absence. We therefore adopted recent proposals to use Bayes factors to compare null and alternate hypotheses. The null hypothesis (H0) here corresponds to the difference between each person׳s EE and FM score being equal for Young and Older groups, whereas the alternate hypothesis, based on Sharon et al.׳s finding, corresponds to the EE–FM difference being greater in Young than Older groups. The latter hypothesis reflects the fact that Sharon et al. found better performance (collapsing across 10 min and 1 week Delay) for EE (*M*=.80 estimated from their Fig. 3) than FM (*M*=.61) in their Controls, but the opposite pattern of better performance for FM (*M*=.64) than EE (*M*=.41) in their Patients. If our Older group are functionally comparable to Sharon et al.׳s patients (albeit with milder amnesia and Hippocampal damage; see below), then we would predict an interaction effect between EE–FM and Age group of up to .42. In fact, the present interaction effect was −.014.

Using the Bayesian approach described by Dienes ((2011); see also http://www.lifesci.sussex.ac.uk/home/Zoltan_Dienes/inference/Bayes.htm), we compared H0 with two alternate hypotheses. Alternate hypothesis H1 was that the plausible size of the above interaction term for the population of young and older ages considered here corresponds to a uniform distribution between a minimum of zero and maximum corresponding to that found by Sharon et al. (i.e., .42). In other words, the advantage of FM over EE for older relative to young people should lie somewhere below that for the extreme case of patients with Hippocampal lesions. In this case, the Bayes Factor favoured the null hypothesis H0 with an evidence ratio of 10.4, i.e, the H0 is approximately 10 times more likely than H1 (a Bayes Factor greater than 3 is often taken as “substantial evidence” for a hypothesis; Jeffreys (1961)). Alternate hypothesis H2 was that the above interaction comes from a half-Gaussian with mode of zero and standard deviation that is half that of the interaction effect found by Sharon et al. (2011). Unlike H1, this hypothesis predicts that interaction values closer to zero are more likely, with the probability of a value as high as that found by Sharon et al. being about 5%. Even with this less extreme alternate hypothesis however, the Bayes Factor was 6.53 times in favour of H0. These analyses support our claim that there is no greater advantage of fast-mapping relative to standard explicit encoding with increasing age.

### 3.6 Multiple regressions

To see whether individual differences in Hippocampal GMV correlated with memory performance, we fit a general linear model (GLM) to the concatenated memory scores. The GLM contained a separate regressor for each task (FM vs. EE) that represented the GMV estimates concatenated across the two groups. We also added regressors of no interest, again separately for each task, to co-vary out differences in TIV and Sex, plus separate regressors for each participant. Modelling all the data in this way allowed us to test for effects of GMV on memory performance that were common to both FM and EE tasks, and to test for GMV effects that differed across tasks.

**Fig. 4:**
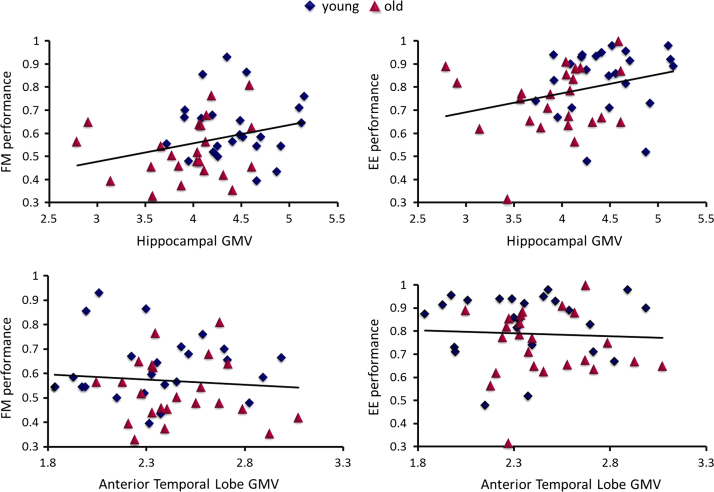
Plots of FM performance (left) and EE performance (right) against grey-matter volume (GMV) estimates for Hippocampus (top panel) and Anterior Temporal Lobe (bottom panel), for young (blue) and Older (red) groups. (For interpretation of the references to colour in this figure legend, the reader is referred to the web version of this article.)

When using the Hippocampal GMV estimates, there was a significant positive relationship between memory scores and Hippocampal volume when averaging across both tasks (*T*(44)=3.61, *p*<.001), but no evidence that this relationship differed between FM and EE tasks (*T*(44)=.22, *p*=.82). Tests of a positive relationship for each task alone (see Fig. 4a) revealed a significant effect in the EE task (*T*(44)=2.31, *p*=.026) and a trend in the FM task (*T*(44)=1.94, *p*=.060).^3^ When we repeated this analysis using the ATL GMV estimates rather than Hippocampal estimates (Fig. 4b), there was no evidence for a relationship with memory performance, either averaging across tasks (*T*(44)=.86, *p*=.39), or for each task separately (*T*(44)<.65, *p*>.51).

Given that our Young and Older groups also differed in Cattell estimates of general intelligence (see Table 1), we fit a further model using Hippocampal GMV estimates, but with an additional regressor for each task that represented IQ (as well as regressors for TIV and Sex, as above). Interestingly, the positive dependence of memory performance (averaged across task) on Hippocampal GMV remained significant, *T*(43)=2.85, *p*=.007,^4^ after the effects of IQ had been accounted for, suggesting that Hippocampal volumes make a contribution to memory that is independent of IQ (Pearson׳s correlation between raw Hippocampal GMV and IQ scores was *R*=.30). There was also a separate contribution from IQ, as evidenced by a significant positive dependence of average memory score on IQ, *T*(43)=5.62, *p*<.001 (and no evidence that this dependence differed for FM vs. EE tasks, *T*(43)=.13, *p*=.90). Finally, we fit a model with an additional regressor that represented Age (i.e., in addition to Hippocampal GMV, IQ, TIV and Sex). In this case, the dependence of average memory performance on Hippocampal GMV no longer reached two-tailed significance, *T*(42)=1.94, *p*=.060 (though IQ and Age continued to make significant independent contributions, *T*(42)=4.98, *p*<.001 and *T*(42)=−3.97, *p*<.001, respectively). Nonetheless, given the strong negative correlation between Age and Hippocampal GMV values (Pearson׳s *R*=−.58), the lack of a significant residual effect of Hippocampal GMV is difficult to interpret, since the shared variance in memory scores could be attributed to either cause, i.e., age or Hippocampal GMV.

## 4 Discussion

Based on a prior report that amnesia owing to acquired Hippocampal damage can be ameliorated through a type of memory encoding called “fast-mapping” (Sharon et al., 2011), we wanted to see whether fast-mapping would also help alleviate memory deficits associated with normal healthy ageing. As expected, our Older group showed worse memory than a Young group (in a standard paired associate learning condition called “explicit encoding”, or EE), together with evidence of smaller Hippocampal volumes than the Young group. We found no evidence however that fast-mapping (FM) reduces this memory impairment in the Older relative to Young group (i.e, no lesser effect of age in the FM than EE condition). Furthermore, we found that Hippocampal volumes predicted memory performance in the FM, as well as EE, condition, suggesting that both tasks were supported by the same medial temporal lobe (MTL) memory system assumed to enable rapid associative learning. We found no evidence, on the other hand, that the volumes of the Anterior Temporal Lobe, a non-MTL cortical region hypothesised to support fast-mapping (Sharon et al., 2011), predicted memory performance in either FM or EE conditions.

One possible reason for the discrepancy between our results and those of Sharon et al. (2011) is that healthy Older people are not “functionally equivalent” to (mild) cases of amnesia following acquired Hippocampal lesions. For example, the age-related shrinking of the Hippocampi observed here (and in many previous studies of ageing, e.g., (Du et al., 2006, Jernigan et al., 2001, Raz et al., 2004, Schuff et al., 1999) may not affect Hippocampal function in the same way that encephalitis, anoxia or surgery did in the four patients studied by Sharon et al. (2011). Alternatively, the simple extent of the Hippocampal damage in our Older group (88% of the Young group׳s Hippocampal volumes on average) may not be sufficient to affect Hippocampal function compared to the greater extent of damage in Sharon et al.׳s patients (which ranged from 35 to 88% of age-matched controls). If fast-mapping is a process that only occurs when “normal” Hippocampally-mediated explicit/episodic encoding is sufficiently impaired (Sharon et al., 2011), then either of the above two factors might explain why we did not see any advantage of fast-mapping for our Older group.

**Supplementary Fig. S1:**
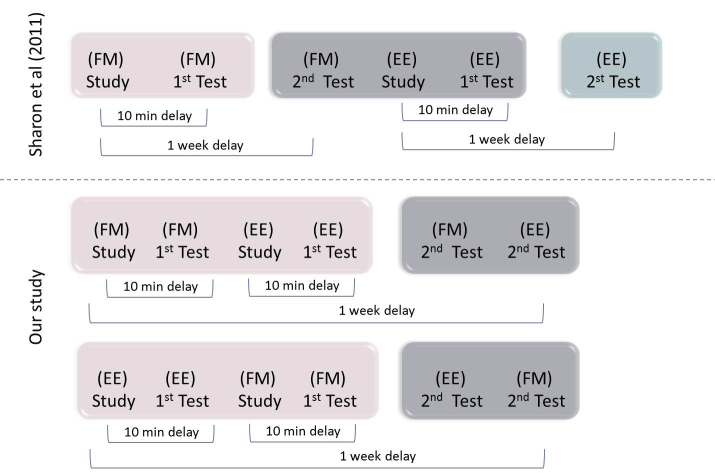
Experimental design showing the task order and schedule of Sharon et al. (2011) compared to the study reported here.

Another possible reason for the discrepancy between our results and those of Sharon et al. (2011) may relate to procedural differences between the experiments. The most obvious of these concern the counterbalancing of stimuli and tasks, and the timing of tasks. Firstly, Sharon et al. (2011) did not counterbalance the assignment of stimuli across tasks (although they did attempt to match stimuli in terms of pre-experimental familiarity ratings). So it remains possible that their differences between FM and EE tasks reflected differences in the ease with which certain stimuli can be encoded, explicitly and/or via fast-mapping. Secondly, Sharon et al. (2011) did not counterbalance the order of tasks, but always ran the FM condition before the EE condition (see Supplementary Fig. S1 for their testing procedure). As fast-mapping is supposed to be an incidental task, one advantage of this order is that the FM condition may be less likely to be contaminated by intentional (explicit) encoding strategies, which might arise if participants have previously performed the EE task. One potential disadvantage however is that the second task (EE task) is associated with more proactive interference than is the first task. We can partially address this issue of task order in the present data, as the task order for the Older group was counterbalanced (See Fig. 2). Our analysis revealed no evidence to suggest that task order matters in this paradigm, at least for the present participants (though it remains possible that the patients in the Sharon et al. study suffered proactive interference to a greater extent than their controls).

A related difference in the order of tasks concerns the fact that our design presented the second test phase after both conditions were tested immediately, while Sharon et al. (2011) ran the two conditions sequentially (see Supplementary Fig. S1). While each task used different stimuli, it is possible that some of retrieval-based interference affected the subsequently delayed test in our design. If this output interference exerted differential effects on FM and EE conditions, this would again predict an interaction with Task Order, which we did not find in our analyses. Nonetheless, we also repeated the ANOVA on the 10 min lag condition only (which would not suffer from this output interference). This showed highly reliable effects of Age group, *F*(1,46)=87.1, *p*<.001, and Task, *F*(1,46)=13.2, *p*<.001, but still no evidence for an interaction between Age and Task, *F*<1.

Sharon et al. interspersed 8 known objects among their 16 unknown objects for each task, with the rationale that this would keep the participants, particularly patients, motivated (even though memory performance was only assessed for the unknown objects). In our study, all 24 studied objects in each task were unfamiliar, potentially increasing our statistical power by 50%, at the risk of de-motivating participants (though any such lack of motivation was not obvious in our healthy participants). This might explain why memory performance of Sharon et al.׳s control group was 7.3% higher on average across all 4 conditions than our Older group, though a more likely explanation is that Sharon et al.׳s controls were younger on average (mean age of 53 years) than our Older group (mean of 66 years). It is conceivable that this higher number of unknown targets in our design compared to Sharon et al.׳s might have required a greater degree of interference resolution at test, potentially driving our observed correlation between Hippocampal GMV and FM performance. It seems unlikely though that Hippocampal-driven interference resolution is critical for the present FM task, because patients suffering severe Hippocampal damage exhibited equivalent (or even slightly better) FM performance relative to healthy controls in Sharon et al. (2011).

A final procedural difference was that Sharon et al. used fixed study trial durations in the EE condition, but self-paced trials in the FM condition. Even though they chose the fixed duration in the EE condition (2380 ms) to match the mean response time in the FM condition, it is possible that the greater (and/or lesser) time spent on some stimuli relative to others caused the differential performance in the FM vs. EE conditions. In our study, we used a fixed duration (of the same 2380 ms as Sharon et al.) for both FM and EE tasks, therefore matching them exactly. Future studies could explore whether this issue of encoding time is important for observing the fast-mapping advantage, and then whether this is a theoretically-important factor or a potential confound.

If the interaction between FM vs. EE and amnesic patients vs. controls reported by Sharon et al. does not owe to a methodological confound (and is not simply a type I error), what does it reflect? The positive correlation between performance of both FM and EE tasks and Hippocampal volume, but not ATL volume, suggests that both tasks rely on similar mechanisms, at least in healthy brains. While this correlation could reflect individual differences other than long-term memory encoding/retrieval (e.g., working memory capacity during the Study phase), it is noteworthy that our correlation with Hippocampal volume remained after covarying out fluid intelligence (IQ), which should capture many such general, non-memory abilities. Further research is needed to address this question.

A recent patient study by Smith et al. (2014; also see Warren & Duff, 2014), however, failed to replicate the results of Sharon et al. (2011). In the Smith et al. (2014) study, seven memory-impaired patients who suffered from either selective Hippocampal or larger MTL lesions completed a FM and EE procedure that was closely matched to the one used by Sharon et al. (2011). The patients were markedly impaired relative to age-matched controls in both EE and FM conditions, i.e. did not show the same advantage of fast-mapping as did the Sharon et al. patients. It is possible that the two patient groups differed in the nature of their memory deficits and/or precise aetiology of brain damage. Although our study never intended to directly replicate Sharon et al.׳s investigation, the failure of Smith et al. (2014) to find FM effects in a similar patient group might indicate that the FM procedure is very sensitive to specific conditions. Interestingly, Smith et al. (2014) also report that patients performed no better than a group of controls who were given the same test, but without previous study of the material, even though both groups performed above chance, which potentially reflects the fact that pre-experimental knowledge allows participants to select the correct response for some of the stimuli used in all of these studies.

We would like to use this observation to suggest an alternative explanation for the discrepancy between Sharon et al.׳s and our findings. This is based on the hypothesis that participants could perform above chance on the 3AFC with the stimuli used here, even if they did not study those stimuli. This hypothesis is based on the fact that many of the names of the novel objects have family resemblance to known object names (e.g., that a “mangosteen” is more likely to be a type of fruit than a type of flower, owing to the morpheme “mango”). If so, the similar performance of patients and controls in Sharon et al.׳s FM condition could have reflected similar inferential processes in both groups based on such pre-experimental knowledge (even if such processes were not conscious). In the EE condition, on the other hand, controls may switch to using their intact anterograde memory to perform much better than in FM conditions. It remains unclear, however, why controls did not also use this intact associative memory in the FM condition. Furthermore, Sharon et al. report recognition performance that was close to chance for control participants who completed a test session without prior FM learning, for stimuli that were presented in Hebrew. Further research will be needed to resolve the discrepancy between these findings.

## Figures and Tables

**Table 1:** Group means (standard deviations in parentheses) for Age, IQ (based on Cattell norms), grey matter volume (GMV) for Hippocampal (Hipp) and Anterior Temporal Lobe (ATL) ROIs and total intracranial volume (TIV).

M (SD)	Young (*N*=24)	Older (*N*=24)
Age/years	26.9	66.0
	(7.4)	(6.3)
Hipp GMV/cm^3^	4.44	3.92
	(.41)	(.49)
ATL GMV/cm^3^	2.36	2.46
	(.31)	(.25)
TIV/cm^3^	1583	1477
	(151)	(110)
IQ/standardised	128	104
	(16)	(14)
Sex (no. of females)	16	14

## Appendix A Supporting information

Supplementary Table T1 List of target trials used by Sharon et al. (2011) compared to target trials tested in the study reported here. Left column shows corresponding target trials tested in both studies. The middle column lists pre-experimental familiar target trials tested by Sharon et al. Our study did not present pre-experimental familiar targets, which we replaced with unfamiliar items (see right column for comparison). The right column lists unfamiliar target trials that differed between the two studies and include the 16 unfamiliar items that were used instead of Sharon et al.׳s familiar target trials.

## Footnotes

1 Note that the Older group were not simply selecting 1 of the 3 alternatives at random in the FM condition, because their performance was significantly higher than 33% [*T*(23)=18.7, *p*<.001]. Nonetheless, true guessing levels may be higher than 33% in this design, since general knowledge about the language (e.g., family resemblance) would allow correct decisions for some of the stimuli even without prior study (e.g., that “mangosteen” sounds more like a fruit than a flower; Fig. 1) – see Sharon et al. (2011) and Supplementary marterial for further discussion.

2 Note that all of the Young group performed the tasks in the same order, of FM then EE (see Section 2), as in Sharon et al. (2011), leading to twice as many participants than in the Older group for this analysis. Nonetheless, exactly the same pattern of significant results emerged with a balanced ANOVA based on the first 12 Young participants.

3 To double-check that these relationships between Hippocampal GMV and performance in FM and EE tasks did not vary with retention interval (10 min vs. 1 week), we split the data by a further Delay factor. The resulting GLM showed a highly significant positive relationship between Hippocampal grey matter at both Short Delay, *T*(132)=2.49, *p*=.007, and Long Delay, *T*(132)=2.63, *p*=.005, when averaging across FM and EE tasks. More importantly, there was no evidence that this Hippocampal–performance relationship at both Delays varied with Task, in that the interaction between Delay and Task was not significant, *T*(132)=−.08, *p*=.46.

4 The same pattern of results remained when FM encoding performance was included as a further covariate of no interest, *T*(42)=2.63, *p*<.05. This finding reinforces the conclusion that Hippocampal volumes predict memory performance in FM conditions, even when accounting for individual variations in processing information during the FM encoding task.
