# Supplementary material for: No evidence that ‘fast-mapping’ benefits novel learning in healthy Older adults
Source: Neuropsychologia. 2014 Jul;60(100):52–9. doi: 10.1016/j.neuropsychologia.2014.05.011 (PMC4115174; doi:10.1016/j.neuropsychologia.2014.05.011)
Supplement: Supplementary file 1 — Supplementary Table T1 List of target trials used by Sharon et al. (2011) compared to target trials tested in the study reported here. Left column shows corresponding target trials tested in both studies. The middle column lists pre-experimental familiar target trials tested by Sharon et al. Our study did not present pre-experimental familiar targets, which we replaced with unfamiliar items (see right column for comparison). The right column lists unfamiliar target trials that differed between the two studies and include the 16 unfamiliar items that were used instead of Sharon et al.׳s familiar target trials. [file mmc2.doc]

| **Identical Unfamiliar Stimuli** | |  | **Familiar Stimuli** |  | **Unidentical Unfamiliar Stimuli** | |
| --- | --- | --- | --- | --- | --- | --- |
| ***Sharon et al*** | ***our study*** |  | ***Sharon et al*** |  | ***Sharon et al*** | ***our study*** |
| Chayote | Chayote |  | Asparagus |  | Aardvark | Anhinga |
| Desman | Desman |  | Carrot |  | Bongo | Banksia |
| Hookoh | Hookoh |  | Coconut |  | Ibis | Cardoon |
| Ignea | Ignea |  | Dog |  | Marmoset | Celtuce |
| Indri | Indri |  | Dolphin |  | Rhubarb | Cherimoya |
| Karela | Karela |  | Eggplant |  | Romanesco | Coati |
| Kobus | Kobus |  | Elephant |  | Sagittarius | Deroptyus |
| Longan | Longan |  | Flamingo |  | Saki | Hoopoe |
| Mangosteen | Mangosteen |  | Giraffe |  | Tersier | Ixora |
| Manroot | Manroot |  | Goat |  | Tomatillo | Jaboticaba |
| Mara | Mara |  | Lion |  |  | Lammergeyer |
| Motmot | Motmot |  | Melon |  |  | Langur |
| Naranjilla | Naranjilla |  | Onion |  |  | Larvatus |
| Numbat | Numbat |  | Penguin |  |  | Loris |
| Pandan | Pandan |  | Pomegranate |  |  | Margay |
| Picroides | Picroides |  | Turtle |  |  | Mesia |
| Rumex | Rumex |  |  |  |  | Monestera |
| Santol | Santol |  |  |  |  | Olm |
| Sapodilla | Sapodilla |  |  |  |  | Pangolin |
| Shipova | Shipova |  |  |  |  | Pepino |
| Tenrec | Tenrec |  |  |  |  | Pitanga |
| Torako | Torako |  |  |  |  | Quetzal |
|  |  |  |  |  |  | Solenodon |
|  |  |  |  |  |  | Soncoya |
|  |  |  |  |  |  | Tahr  Trilium |
